# Quantifying the effects of far-red light on lettuce photosynthesis and growth using a 3D modelling approach

**DOI:** 10.3389/fpls.2024.1492431

**Published:** 2024-11-29

**Authors:** Jiawei Li, Yuanyuan Li, Yunke Chen, Shipu Xu, Xue Wu, Cuinan Wu, Ningyi Zhang, Kai Cao

**Affiliations:** ^1^ School of Agricultural Engineering, Jiangsu University, Zhenjiang, Jiangsu, China; ^2^ Key Laboratory of Agricultural Engineering in the Middle and Lower Reaches of Yangtze River, Ministry of Agriculture, Institute of Agricultural Facilities and Equipment, Jiangsu Academy of Agricultural Sciences, Nanjing, Jiangsu, China; ^3^ Horticulture and Product Physiology, Department of Plant Sciences, Wageningen University and Research, Wageningen, Netherlands; ^4^ Key Laboratory of Smart Agricultural Technology (Yangtze River Delta), Ministry of Agriculture and Rural Affairs, Agricultural Science and Technology Information Institute, Shanghai Academy of Agricultural Sciences, Shanghai, China; ^5^ College of Horticulture, Nanjing Agricultural University, Nanjing, China

**Keywords:** vertical farms, far-red light, 3D plant model, light interception, photosynthesis

## Abstract

In vertical farms, the supplementation of far-red light has been widely applied to regulate plant growth and development. However, the relative contribution of far-red to photosynthesis and plant growth in indoor production systems is not sufficiently quantified. This study quantify the photosynthesis and growth responses under different levels of supplemental far-red in lettuce using a 3D modelling approach. Lettuce were cultivated under either white light or red to far-red (R:FR) ratio of 1.6 or 0.8. Measurements of plant morphological traits, leaf photosynthesis, and organ fresh and dry mass were taken and the 3D modelling approach was used to simulate plant photosynthesis and biomass accumulation. Results showed that leaf elevation angle, leaf expansion rate, and plant height significantly increased at each growth stage as the R:FR ratio decreased. Far-red light also promoted plant growth, leading to an increase in the dry and fresh weight of lettuce throughout the entire growth period. However, plants cultivated at low R:FR showed reduced maximum Rubisco carboxylation rate and maximum electron transport rate, which indicated that far-red light reduced the photosynthetic capacity in lettuce. Nevertheless, 3D model simulations demonstrated that plants exposed to enhanced far-red light exhibited increased light interception and whole-plant photosynthesis. The integrated analysis of photosynthetic parameters and plant morphological changes on the photosynthetic rate of the whole plant indicated that the positive effects of plant morphological changes outweighed the negative impacts of photosynthetic parameters. These results implied that far-red light-induced morphological changes enhanced light interception and whole-plant photosynthesis, thereby increased lettuce yield.

## 1 Introduction

Vertical farming involves growing plants in stacked layers in indoor environment, where temperature, light, humidity, and other environmental factors are precisely controlled to maximize growth and yield. This novel indoor production system brings many advantages – e.g. shortening growth cycle, increasing yield production per unit land area, and reducing water usage by drainage circulation – which makes it an ideal solution for city farming (Al-Chalabi, 2015; Al-Kodmany, 2018; SharathKumar et al., 2020). As a model of resource-efficient agricultural production system, vertical farm largely increases agricultural resource use efficiencies and decreases transportational costs by bringing the production system closer to the consuming site. Recently, many research have focused on impacts of environmental conditions – e.g. light, water, and nutrient – on growth and production of such crops as watercress and lettuce in indoor farming systems (Qian et al., 2022; Carotti et al., 2023). Among those ambient conditions, light environment has received special attention due to that the artificial lighting used in vertical farms allows to manipulate not only light intensity and photoperiod but also the light spectrum for enhancing plant growth and development, yield, and product quality (Chen et al., 2018; Izzo et al., 2020; Liu and Van Iersel, 2021). Among different colors of the supplemental light, adding far-red has been found to increase crop production. For example, wheat yield significantly increased and the growth cycle was notably shortened under additional far-red treatment (Dreccer et al., 2022); in tomato, supplemental far-red has been found to increase fruit weight via enhancing dry matter allocation to the fruit (Ji et al., 2019).

Far-red affects plant growth mainly via two aspects – changes in plant morphology and leaf photosynthesis. On the one hand, far-red triggers shade avoidance syndromes in numerous species. Typical shade avoidance responses include changes in leaf orientation, stem elongation, and increased peduncle length (Ballaré and Pierik, 2017). Some species also exhibit increases in leaf area expansion under supplemental far-red (Park and Runkle, 2017). Those morphological changes potentially increase plant light capture under competitive environment. For example, FR light has been found to promote tomato development via increasing light interception (Kalaitzoglou et al., 2019; Kang et al., 2023). On the other hand, far-red light can lead to a decrease in photosynthetic rate, as it reduces stomatal conductance and photosynthetic efficiency within some crops such as kale (Kang et al., 2023). Interestingly, given the negative impact of far-red on photosynthetic physiology, many studies showed positive effects of additional far-red light on crop yield across various controlled-environment agricultural setups, such as greenhouses and vertical farms (Zhen and van Iersel, 2017; Meng and Runkle, 2019; Ji et al., 2020). It is possible that the level of positive effects of morphological changes on light capture induced by far-red exceeds the level of negative impacts of far-red on leaf photosynthesis, resulting in an overall positive impact on plant growth. Quantitatively analyzing the relative contribution of plant morphology and leaf photosynthesis on plant growth under far-red could help to better understand the productivity of indoor plant production systems including vertical farms for light recipe optimizations, and to identify important traits for breeding special varieties for vertical farms.

Plant 3D architectural models can integrate with various physiological sub-models to quantitatively analyze plant responses to environmental factors. Most previous studies measured plant light interception using 3D digitized canopy or top-down photography methods (Hui et al., 2018; Tang et al., 2019; Xiao et al., 2023). Nevertheless, these methods simplify the actual plant growth morphology and neglect light penetration through leaves. This study employs a method of ray-tracing approach on scanning representations to optimize estimations of light interception. Ray-tracing technology has been widely used to assess imaging performance, estimate plant light interception, and simulate electrical lighting in plant factories (Van der Zande et al., 2011; Shin et al., 2021; Hwang et al., 2023). Previous studies have shown that the impact of Ultraviolet-B radiation on kale plants and the effects of LED arrangement and installation angle in indoor plant production systems using ray-tracing simulation methods (Yoon et al., 2022a, b). These studies suggest the reliability of such type of approach in modeling light effects in vertical farms.

Lettuce (*Lactuca sativa* L., cv. *Italian*) is widely cultivated as leafy greens worldwide and is one of the major vegetables cultivated under protected conditions in China. Given its relatively small size, short production cycle, and high demand for fresh consuming, there is an increasing interest in growing lettuce in vertical farms. Previously, enhanced far-red light has been found to increase lettuce yield (Kong and Nemali, 2021). However, the relative contributions of far-red-induced changes in plant morphology and leaf photosynthesis on lettuce production have not yet been quantified. The study aimed to explore how alterations in plant morphology and leaf photosynthesis influence lettuce growth and yield under supplemental far-red light, offering valuable insights for optimizing light strategies in vertical farm lettuce production. To this end, an experiment with different levels of supplemental far-red was conducted on lettuce. Measurements were taken for plant structure, leaf photosynthesis, as well as the fresh and dry mass of the plants. A 3D lettuce plant model was developed for quantifying light interception, photosynthesis, and biomass production.

## 2 Materials and methods

### 2.1 Plant material and growth environment

Seeds of Lettuce (Lactuca sativa L., cv. Italian) were planted and sprouted on sponge cubes (Good Earth Agriculture Company, Hunan, China). The young lettuce plants were cultivated under the illumination of fluorescent lights (SMD2835, Guangzhou Inled Lighting Technology Company, Guangzhou, China), which provided a photosynthetic photon flux density (PPFD) measuring 250 μmol m^–2^ s ^–1^. In the propagation phase, Hoagland nutrient solution (pH: ~ 6.8; EC: 1.8 – 2.2 mS cm^-1^) was used for cultivation. After two weeks of sowing, the young plants were established within vertical agriculture setups which had three layers (volume of each layer: 130×60×30 cm). In the vertical farm, the environmental temperature was sustained at 25°C, with the humidity level targeted between 60–70%, and the photoperiod was established at 16 hours for the light phase.

**Figure 1:**
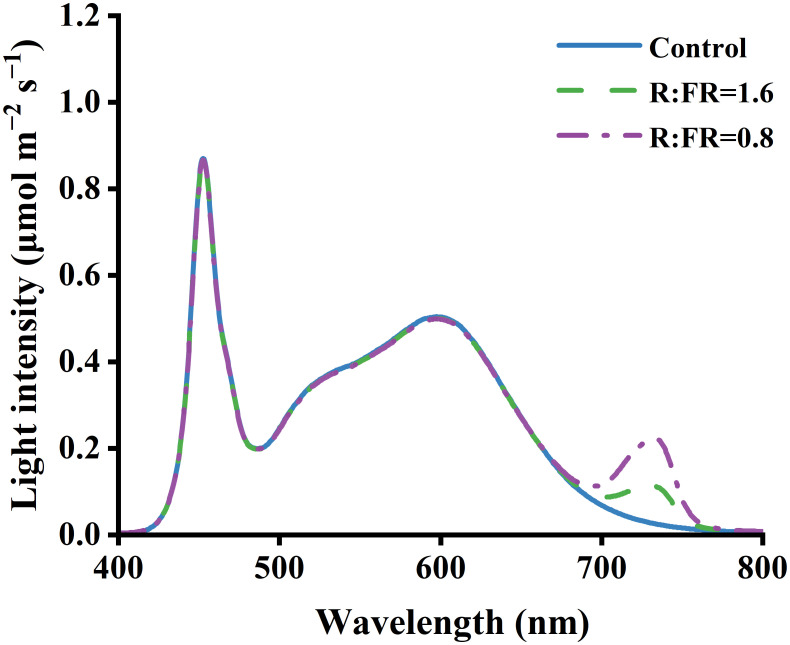
Light spectra of treatments under R:FR ratio of 6.4, 1.6 and 0.8.

In total there were three light treatments, including a treatment with white light and two treatments with supplemental far-red LEDs. A background PPFD of 250 ± 10 μmol m^–2^ s^–1^ from white and far-red LEDs was present in three conditions. The PPFD was measured as the cumulative flux from 400 to 800 nanometers (**Table 1**). The white light condition served as a control group. In contrast, the other two treatments received additional far-red light at distinct intensities supplied by LEDs. These treatments had R:FR ratios of 1.6 and 0.8, which will be referred to as R:FR(1.6) and R:FR(0.8) in subsequent discussions. The spectral irradiance was quantified utilizing a spectroradiometer (PS-300, Apogee Instruments 136 Inc., Logan, UT, USA) (**Figure 1**).

**Table 1:** List of spectral characteristics under different far-red (FR) fractions.

Treatment	Blue (μmol m^−2^ s^−1^)	Green (μmol m^−2^ s^−1^)	Red (μmol m^−2^ s^−1^)	Far red (μmol m^−2^ s^−1^)	PPFD (μmol m^−2^ s^−1^)	R:FR ratio
Control	52	84	113	18	246	6.4
R:FR=1.6	52	84	113	71	246	1.6
R:FR=0.8	52	84	113	141	246	0.8

### 2.2 Measurements of plant morphology, biomass, and leaf optical property

The fresh and dry weights of leaves and roots, plant height, and total leaf area were measured in six plants per treatment on 7, 14 and 21 days after treatment. Organ dry weight was measured by drying the organ in an oven at 70°C for three days. To measure the leaf area, photos of individual lettuce plants were taken at first. The leaves were laid flat and photos were taken from directly above at a 90-degree angle. Then the photos were analyzed in an image analysis software (ImageJ 1.49, National Institutes of Health, Bethesda, MD, USA). The light transmittance, reflectance, and absorption of lettuce leaves was measured using a spectroradiometer and cosine corrector (PS-300, Apogee Instruments 136 Inc., Logan, UT, USA) in the 400–800 nm. First, standard reference materials were used to perform white and dark point calibration. Next, spectroradiometer was set to transmittance mode, and adjusted the wavelength range to 400-800 nm. At last, the light source was positioned on one side of the sample and the spectroradiometer probe on the other side. A similar approach was adopted to measure reflectance. For each treatment group, the transmittance and reflectance of lettuce leaves were assessed on three leaves chosen at random. Subsequently, leaf absorptance was determined by subtracting the transmittance and reflectance from 100%.

### 2.3 Leaf gas exchange and chlorophyll fluorescence measurements

Three plants in each treatment were chosen to conduct leaf gas exchange and chlorophyll fluorescence measurements. The third leaf counting from the plant top was selected to measure photosynthetic light response curve, photosynthetic rates versus intercellular CO_2_ concentrations (*A/C_i_*) curve, instantaneous leaf photosynthetic rates with a clear-top chamber, and chlorophyll fluorescence.

Ten days after treatment, the photosynthetic light response and the *A/C_i_* curves were obtained using a portable gas exchange system (LI-6800XT, Li-Cor, Lincoln, NE, USA), which was equipped with LED light sources comprising predominantly red (90%) and a smaller proportion of blue (10%) light (6800–02B, Li-Cor). During the measurements, leaf temperature was controlled at 25°C and the relative humidity was maintained at 60%. The combined gas exchange and chlorophyll fluorescence measurements were conducted. Leaves were first dark-acclimated in the chamber for 20 minutes while the chamber light source was off. Subsequently, a saturating light pulse was applied using the rectangular flash at 16000 μmol m^-2^ s^-1^ PPFD to determine the maximum chlorophyll fluorescence (*Fm*). Following this measurement, leaves were light-acclimated for 15 min under 1800 μmol m^–2^ s ^–1^ PPFD, with ambient CO_2_ concentration (*C*_a_) being kept at 400 ppm. Light response curves were measured by lowering the light intensity as the following steps: 1800, 1500, 1200, 900, 600, 300, 200, 150, 100, 70, 50, 30, and 0 μmol m^–2^ s ^–1^ PPFD. The chlorophyll fluorescence data *Ф*_2_ were obtained along with the recording of light response curves. After that, light intensity was switched back to 1800 μmol m^–2^ s ^–1^ for 20 min for acclimation, and *A/C_i_* curves were measured by changing the *C*_a_ as the following steps: 400, 300, 200, 100, 50, 75, 100, 125, 150, 200, 300, 400, 500, 600, 800, 1000, and 1200 ppm. Each light step or *C*_a_ step was set to 3 minutes.

Instantaneous leaf net photosynthetic rates under each light treatment were measured with a clear-top chamber (6800–08, Li-Cor) equipped on LI-6800. The leaf was put in the clear-top chamber for 15 min for acclimation of the local light environment from the treatment. Then, gas exchange data were logged three times for each treatment, and the average values were recorded. During the measurements, leaf temperature, relative humidity, and *C_a_* were respectively set at 25°C, 60% and 400 ppm.

The leaf photosynthetic parameters were derived by applying the biochemical photosynthesis model of Farquhar, von Caemmerer, and Berry (the FvCB model) to the combined data from gas exchange and chlorophyll fluorescence measurements (Yin et al., 2009) using an Excel tool (Bellasio et al., 2016):


(1) $$\begin{array}{c} A=\min\left(A_{c},A_{j}\right) \end{array}$$

(2) $$\begin{array}{c} A_{c}\;or\;A_{j}=\left(\frac{\left(C_{c}-\Gamma^{*}\right)x_{1}}{C_{c}+x_{2}}\right)-R_{d} \end{array}$$

(3) $$\begin{array}{c} x_{1}=\{\begin{array}{c} \;V_{cmax}\;\;for\;A_{c}\\ \frac{J}{4}\;\;\;\;\;for\;A_{j} \end{array} \end{array}$$

(4) $$\begin{array}{c} x_{2}=\{\begin{array}{c} K_{mc}\left(1+\frac{O}{K_{mo}}\right)\;for\;A_{C}\\ 2\Gamma^{*}\;for\;A_{j} \end{array} \end{array}$$

where *A* is leaf net assimilation rate (μmol CO_2_ m^-2^ s^-1^); *A_c_* and *A_j_* are leaf net assimilation rates limited by ribulose biphosphate-carboxylase-oxygenase (Rubisco) activity and electron transport rate, respectively; *R_d_* is non-photorespiratory respiration rate (μmol CO_2_ m^-2^ s^-1^); *V_cmax_* is the maximum rate of carboxylation capacity (μmol CO_2_ m^-2^ s^-1^); *C_c_* is CO_2_ concentration in the chloroplast (μmol·mol^-1^); Γ^*^ is CO_2_ compensation point (μmol mol^-1^); *K_mc_* and *K_mo_* are Michaelis–Menten constants of Rubisco for CO_2_ and O_2_ (μmol mol^-1^), respectively, (estimated by fitting the’full Farquhar model’ as developed by (Ethier and Livingston, 2004) to the Rubisco limited part of the *A*/*C*_i_ curve); *O* is oxygen concentration (μmol·mol^-1^).

To convert chlorophyll fluorescence data *Ф*_2_ into apparent electron transport rate *J*, a calibration was done by linear repression plot of A against (*I_inc_Ф_2_*/4) utilizing the information gathered under non-photorespiratory conditions (**Supplementary Figure S1**). The slope s of this linear regression was used as a calibration factor to convert *Ф*_2_ into *J* using Equation 5:


(5) $$\begin{array}{c} J=s\times I_{inc}\times \Phi_{2} \end{array}$$

where *I_inc_* is photosynthetic photon flux density (μmol m^-2^ s^-1^).

The obtained *J* under each light step was then fitted to Equation 6 to estimate electron transport relevant parameters:


(6) $$\begin{array}{c} J=\frac{K_{2\left(ll\right)}I_{inc}+J_{max}-\sqrt{{\left(K_{2\left(ll\right)}I_{inc}+J_{\text{max}}\right)}^{2}-4\theta J_{\text{max}}K_{2\left(ll\right)}I_{inc}}}{2\theta } \end{array}$$

where *J_max_* is maximum electron transport rate (μmol CO_2_ m^-2^ s^-1^); *θ* is curvature of light response of *J* (dimensionless); and *K_2(ll)_* is initial quantum yield for electron transport (dimensionless).

The FvCB model requires *C_c_* as an input, which needs mesophyll conductance *g_m_* to convert *C_i_* obtained from the gas exchange measurements into *C_c_*:


(7) $$\begin{array}{c} C_{c}=C_{i}-\frac{A}{g_{m}} \end{array}$$

By replacing Equation 7 with the *C_c_* in the FvCB model, *g_m_* can be estimated by fitting the gas exchange data with Equations 1–4.

### 2.4 3D model development and simulations of plant light capture

**Figure 2:**
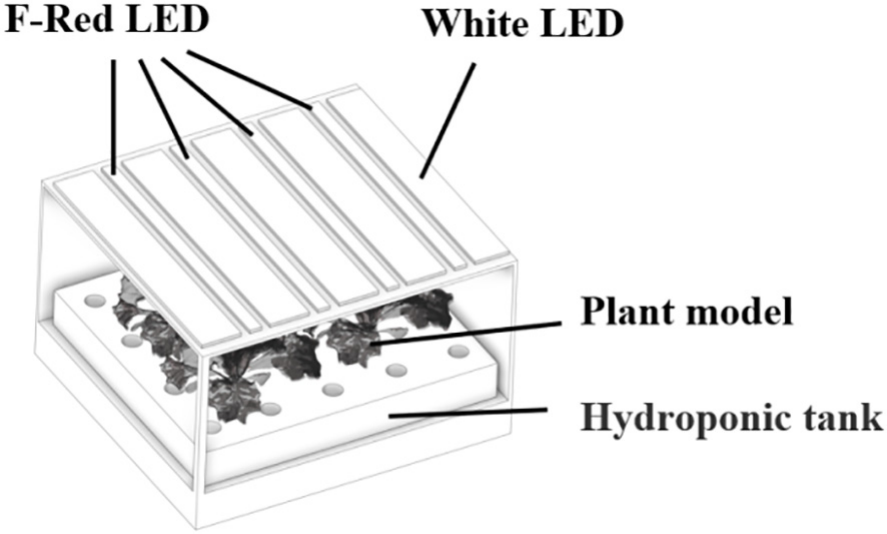
The simulated environment was comprised of a three-dimensional representation of the growth chamber, lighting fixtures, and plant models.

The lettuce plants were scanned by a high-resolution 3D scanner to reconstruct their 3D structure (3DSHandy-7XBS, Shanghai Digital Manufacturing Corp, Shanghai, China). Three plants in each treatment were chosen to conduct the 3D scanning. The measurement rate of the scanner was set at 480,000 times per second and the resolution of the scanner was set at 0.05 mm. After collecting the marker points, the scanning software generated a mesh based on the grid resolution specified. Then, the point cloud data from the 3D scan were converted into a solid model after being processed by the scanning software (Vxelement, CREAFORM, USA). The flawed 3D models were imported into reverse engineering software (Geomagic Design X, 3D Systems, Rock Hill, SC, USA) for noise reduction, mesh repair, and edge sharpening. Then, fixed models were reconstructed in 3D CAD software (Solidworks, Dassault Systems, VelizyVillacoublay, France). Meanwhile, the chamber and light source models were assembled according to the actual dimensions measured from the experiment (**Figure 2**).

A ray-tracing module (Optisworks, OPTIS Inc., La Farlede, France),which had the Monte-Carlo algorithm to calculate trajectories of the rays, was used for the ray tracing simulation (Shin et al., 2021). In this simulation, quantity of rays was established at 2 billion rays. The simulation utilized ray tracing to directly calculate the light interception on the leaf surface. Furthermore, this module provides the output of light intensity at each point cloud from the 3D scanning and automatically calculates the relative frequency of the occurrence of each light intensity value (Kim et al., 2020). The distribution of the relative frequency of each light intensity value provides an evaluation of the light distribution pattern in the scanned object – i.e. the lettuce plant in this study. To verify the accuracy of light simulation, the light intensity was measured using a spectroradiometer (PS-300, Apogee Instruments 136 Inc., Logan, UT, USA) at 0, 10, 20 cm from the floor in an empty growth chamber, with 16 points measured at each height. Then, the simulated light intensities and the measured ones were compared. The measured light intensities reaching the leaf surface were employed to estimate photosynthetic rates.

### 2.5 Calculation of canopy gross photosynthetic rate

The simulation outcomes comprised the point cloud data of the 3D model, detailing the (x, y, z) coordinates, along with the light interception (μmol m^-2^ s^-1^) that was converted to absorbed PPFD, considering the spectral distribution which corresponded to absorption per nanometer (Kim et al., 2020). The i-th point cloud’s (*A_i_*) photosynthetic rate was determined using Equation 8:


(8) $$\begin{array}{c} A_{n,i}=\min\left(A_{c,i},A_{j,i}\right) \end{array}$$

*A*_n_ was calculated by Equation 9:


(9) $$\begin{array}{c} A_{n}=\frac{\sum_{i=1}^{n}(A_{i}\times OA_{i})}{LA} \end{array}$$

where *OA_i_* (m^−2^) represents the area occupied by a point cloud. The variables *n* and *LA* (m^−2^) denote the total number of points and the overall leaf area, respectively. These quantities are subject to variation in accordance with the dimensions of the 3D model.

### 2.6 Simulation of plant biomass production

Given that plant growth conditions – e.g. temperature, humidity, and CO_2_ – in the vertical farm were kept at constant, plant growth was assumed to linearly correlate with time. The linear correlations between plant parameters and time for each lighting condition were measured using the experimentally derived values (**Supplementary Figure S2**). Canopy gross photosynthesis was calculated as (Zhen and Bugbee, 2020):


(10) $$\begin{array}{c} A_{gross}=\left(A\times \;16h-\left|R_{dark}\right|\times \;8h\right)\times y_{leaf\;area}\;cm^{2}\times 30g\;mol^{-1} \end{array}$$

where *A_gross_* is the canopy gross photosynthesis; *A* is the net photosynthesis rate; **|** *R_dark_* **|** was the absolute value of dark respiration; 16 was the light period in hours, and 8 was the dark period in hours; y_leaf area_ was the leaf area; and 30 represents grams dry mass per mole of CO_2_ assimilated, assuming a carbon content of 0.4 g g^−1^ in plant tissues.

The plant dry weight was simulated based on the approach in the SUCROS model, which assumed that the daily carbon allocation is divided between the production (assimilation) and utilization (maintenance and growth) processes. The plant biomass production was calculated as (Goudriaan and Van Laar, 2012):


(11) $$\begin{array}{c} \;Y=\left(A_{gross}-R_{maint}-R_{growth}\right)\times \varphi \times d \end{array}$$

where *Y* is the plant dry weight; *R_maint_* and *R_growth_* is the respiration of maintenance and the respiration of growth, respectively, which were fixed at 0.0205 and 0.8941 (De Vries et al., 1983); *φ* is the conversion factor which were fixed at 0.170 (Amthor, 2000); and *d* was the day after treatment.

### 2.7 Quantifying the level of contribution of individual traits to light capture and plant photosynthesis

The effect of single trait responses on the fraction of light capture or plant photosynthesis was quantified by varying a single parameter value of plant architecture or photosynthesis at a time (Zhang et al., 2020):


(12) $$\begin{array}{c} E=\frac{D_{p}-D}{D} \end{array}$$

where *E* represents the proportional impact of trait response on light interception or photosynthesis; *D*_p_ denotes the light interception or photosynthesis determined using the parameter from the low red to far-red ratio treatments; and *D* represents the light interception or photosynthesis calculated with the parameter from the white light condition.

### 2.8 Statistical analysis

All experimental data were managed and evaluated using SPSS 26.0 (IBM, Armonk, NY, USA) and Origin 2021 software (Northampton, MA, USA). A one-way ANOVA followed by Duncan’s multiple range test at a significance level of p< 0.05 was employed to identify significant differences among the treatment groups on corresponding days.

## 3 Results

### 3.1 Effects of R:FR ratios on plant morphology and biomass

**Figure 3:**
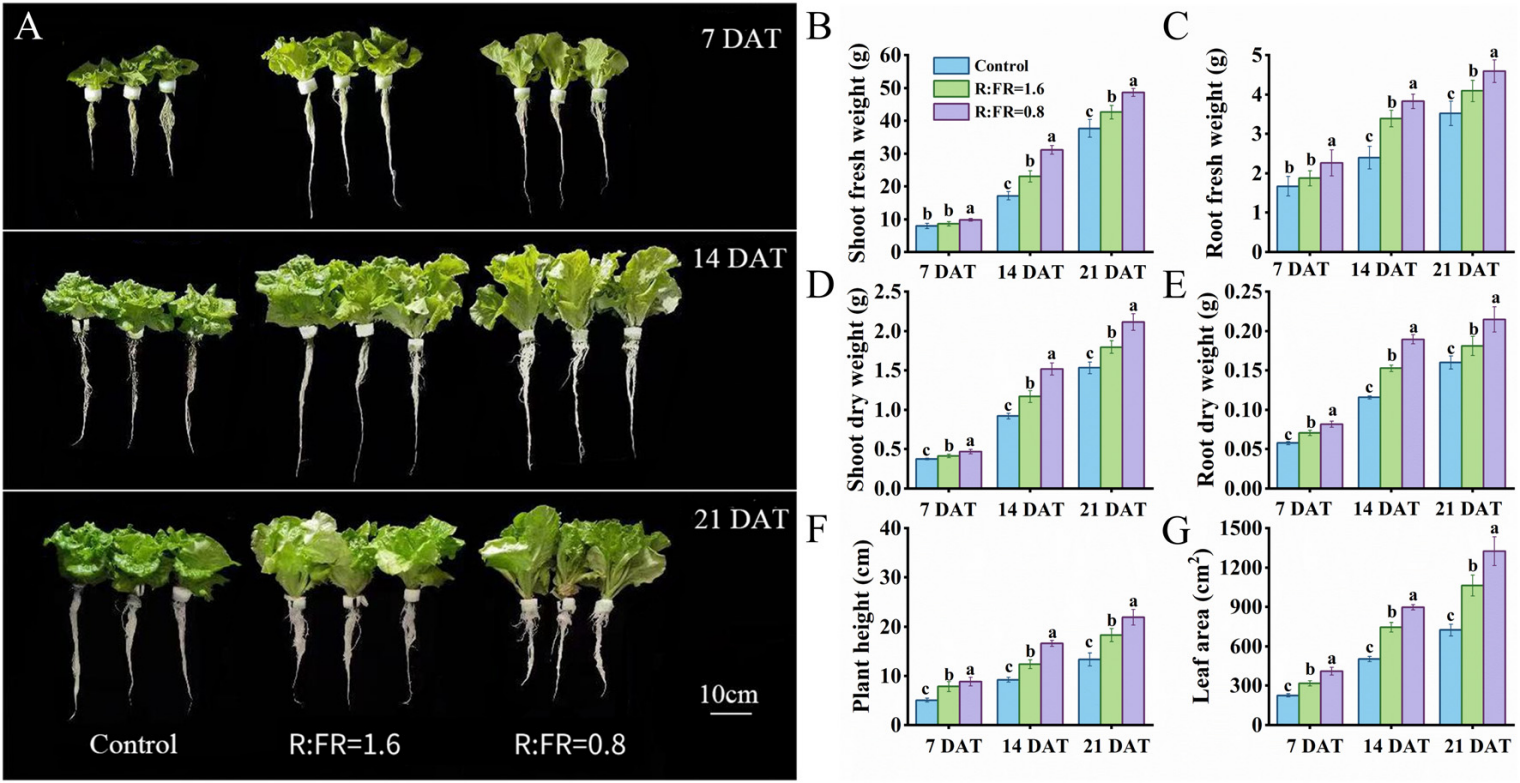
The phenotypes picture for 7, 14 and 21 days after treatment **(A)**. Fresh weight, dry weight, plant height and leaf area of lettuce plants in response of different R:FR ratio light conditions **(B–G)**. The vertical bars indicate SD; n = 6. Different letters at the top of the histogram represent significant difference.

Plant morphology showed substantial differences between the three treatments (**Figure 3A**). The fresh and dry weights of both shoot and root increased with the decreasing of R:FR (**Figures 3B-E**). After 21 days of treatment, the shoot fresh weights of R:FR(1.6) and R:FR(0.8) significantly increased by 11.7% and 25.8%, respectively, compared to plants grown under white light, and the shoot dry weights of R:FR(1.6) and R:FR(0.8) were significantly increased by 17.2% and 37.8%, respectively (**Figures 3C, E**). Plant height and leaf area were also significantly increased with the decreasing of R:FR. After 14 days of treatment, the plant height and leaf area in FR light treatments were significantly increased by approximately 43.9% and 78.1%, respectively, compared to plants grown under white light, and after 21 days of treatment, these values exhibited a further significant increase to 48.1% and 83.1%, respectively (**Figures 3F, G**).

### 3.2 Effects of R:FR ratios on leaf photosynthetic efficiency and optical properties

**Figure 4:**
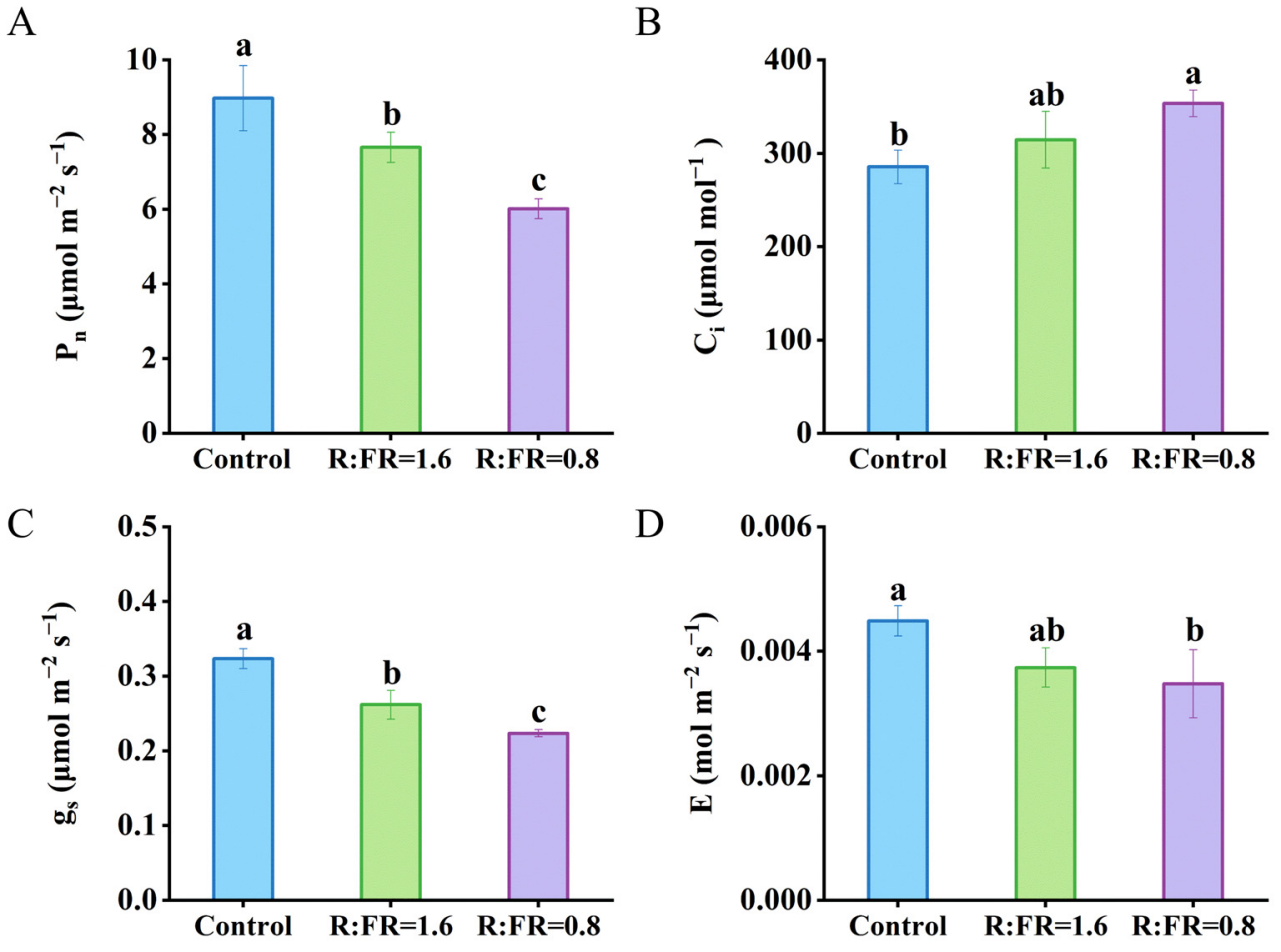
Effects of different R:FR ratio treatments on the photosynthetic rate (P_n_) **(A)**, intercellular CO_2_ concentration (C_i_) **(B)**, stomatal conductance (g_s_) **(C)** and transpiration rate (E) **(D)** of lettuce plants. The vertical bars indicate SD; n = 3. Different letters at the top of the histogram represent significant differences (p < 0.05).

Instantaneous leaf net photosynthesis rate, stomatal conductance, and transpiration rate measured under growth conditions all decreased with the lowering of R:FR (**Figure 4**). Leaf photosynthetic rates under R:FR ratios of 1.6 and 0.8 were significantly decreased by 14.7% and 33.1%, respectively, compared to plants grown under white light (**Figure 4A**). The intercellular CO_2_ concentration increased with the lowering of R:FR (**Figure 4B**). The stomatal conductance was significantly reduced by 19.1% and 30.9% respectively in the R:FR(1.6) and R:FR(0.8) compared to plants grown under white light (**Figure 4C**). The transpiration rate decreased with the lowering of R:FR (**Figure 4D**).

Leaf photosynthetic parameters were significantly affected by the light treatments. *J*_max_ significantly reduced under the R:FR(1.6) and R:FR(0.8) light treatment by 17.1% and 31.9% compared to *J_max_* of plants grown under white light, and *V*_c_ *_max_* significantly reduced by 17.1% and 31.9% respectively (**Table 2**). Moreover, *R_d_* showed a decreasing trend in the R:FR(1.6) and R:FR(0.8) by 22.1% and 39.3%, respectively, compared to *R_d_* obtained under white light (**Table 2**). The light treatments did not have a significant impact on the curvature *θ*. (**Table 2**). Compared to the white light treatment, *g_m_* significantly decreased respectively by 17.4% and 29.8% under the R:FR(1.6) and R:FR(0.8) light treatment. *K_2ll_* decreased with the increasing of far-red light intensity (**Table 2**).

**Table 2:** FvCB model parameters of lettuces under different R:FR ratio treatments.

Treatment	*J_max_*	*V_cmax_*	*θ*	Γ*	*R_d_*	*g_m_*	*K_2(ll)_*
Control	157.2 ± 15.8 a	82.5 ± 6.2 a	0.936 ± 0.114 a	42.1 ± 2.9 a	1.251 ± 0.051 a	0.321 ± 0.029 a	0.257 ± 0.019 a
R:FR=1.6	130.4 ± 9.7 b	71.9 ± 4.3 b	0.913 ± 0.028 a	33.1 ± 3.6 b	0.974 ± 0.131 b	0.265 ± 0.011 b	0.237 ± 0.013 ab
R:FR=0.8	107.1 ± 6.7 c	51.5 ± 3.8 c	0.892 ± 0.054 a	30.5 ± 3.1 b	0.759 ± 0.034 c	0.225 ± 0.016 c	0.218 ± 0.08 b

*J_max_*, Maximum value of *J* under saturated light; *V_cmax_*, maximum rate of Rubisco activity-limited carboxylation; *θ*, curvature of light response of *J*; Γ^*^, CO_2_ compensation point; *R_d_*, non-photorespiratory respiration rate; *g_m_*, mesophyll diffusion conductance; *K_2(ll)_*, initial quantum yield for electron transport (dimensionless).

Letters indicate significant differences (P< 0.05, n = 3).

Both leaf transmittance and reflectance increased with the decreasing of R:FR. Leaf transmittance under R:FR ratios of 1.6 and 0.8 respectively increased by 23.8% and 29.9%, and leaf reflectance under R:FR ratios of 1.6 and 0.8 respectively increased by 14.1% and 28.1%. Consequently, leaf absorptance under R:FR ratios of 1.6 and 0.8 respectively decreased by 5.3% and 8.2%, compared to leaf absorptance of control (**Supplementary Figure S3**).

### 3.3 Simulations of plant light capture, photosynthesis, and dry mass production

**Figure 5:**
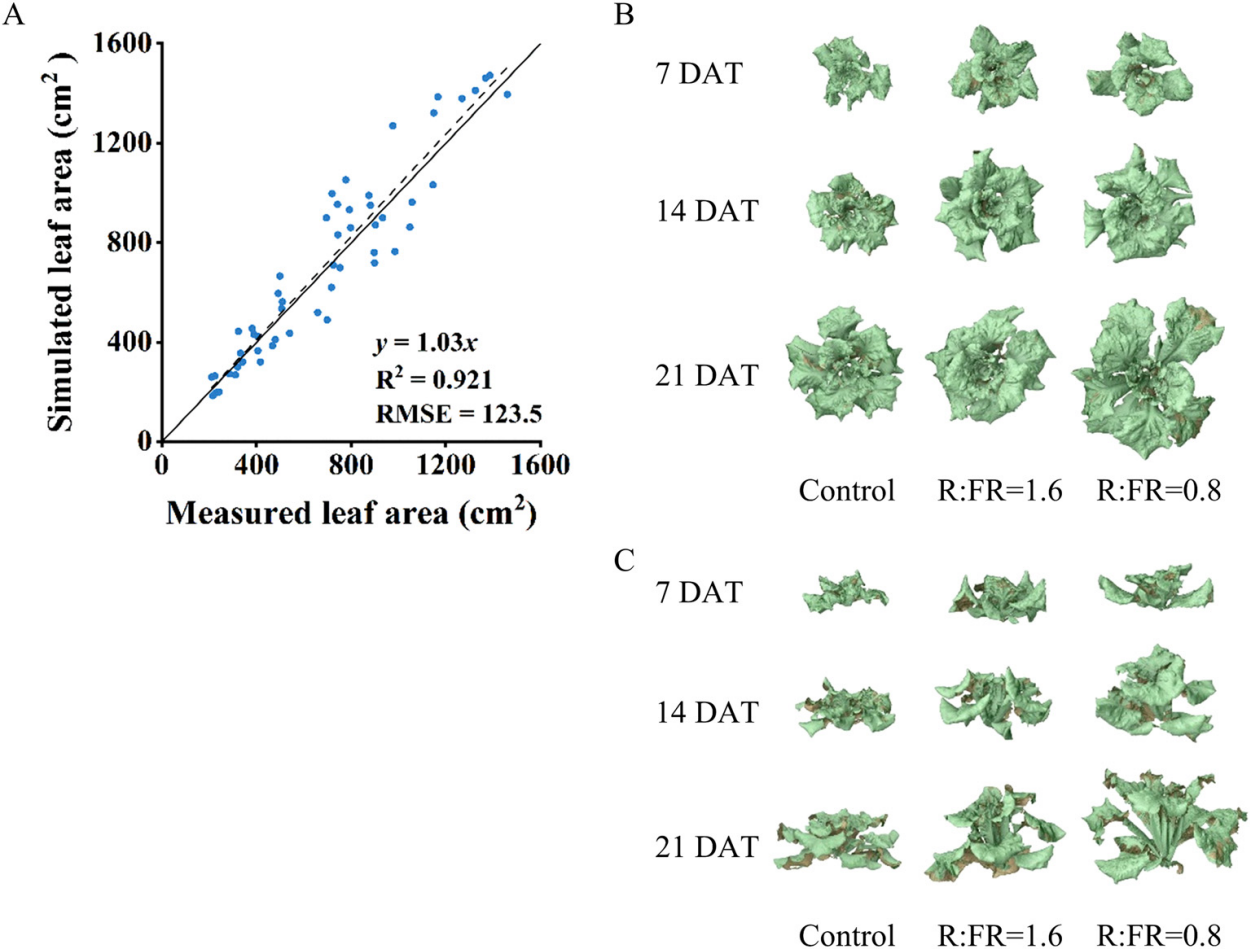
Comparison between measured and simulated leaf areas **(A)**. The top view **(B)** and front view **(C)** of 3D models for lettuce plants at 7, 14, 21 days after different R:FR ratio light conditions. Eight lettuce plants of each treatment were used for measurement. The solid line represents a fitted linear and the dashed line represents a 1:1 relationship.

**Figure 6:**
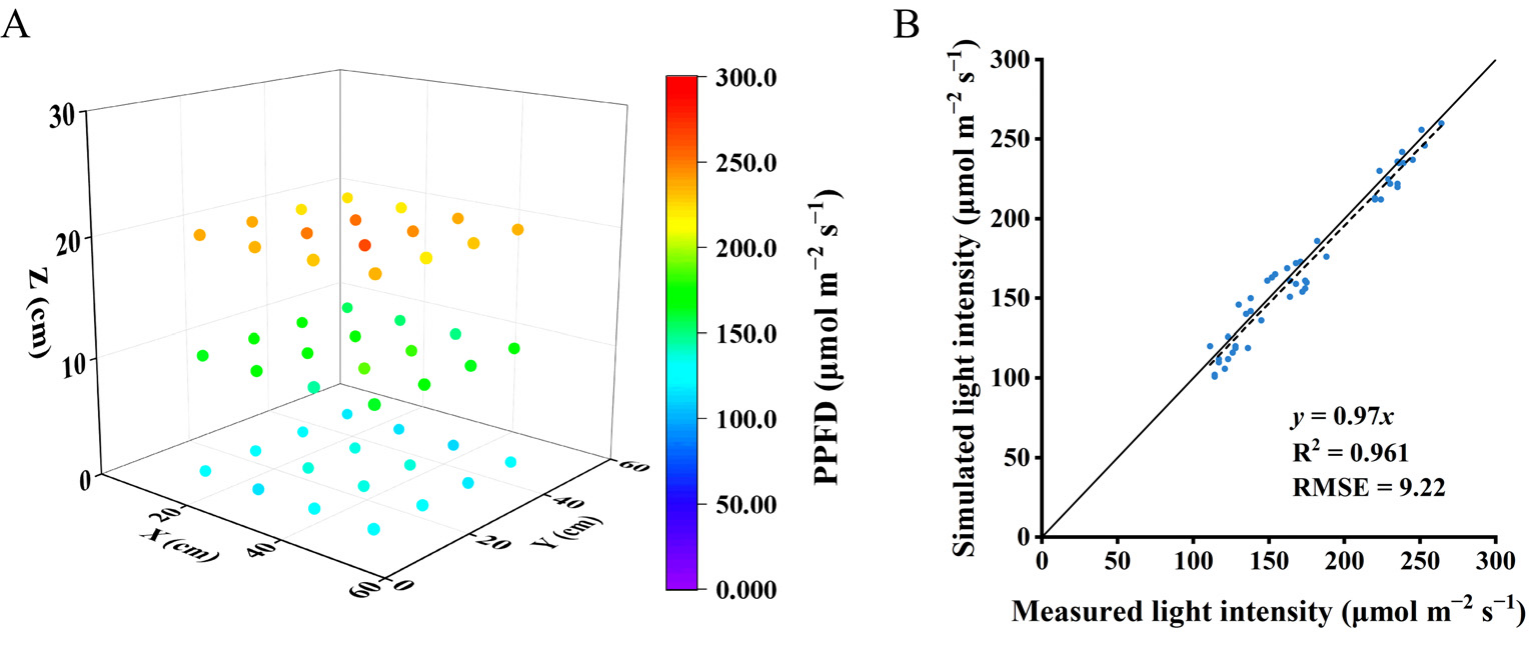
Light intensities within the vacant chamber were gauged at various elevations starting from the ground level at 0, 10, 20 and 30 cm with sixteen horizontal positions at each height **(A)**. Evaluating the concordance between actual and simulated light intensities within the empty chamber **(B)**. The solid line represents a fitted linear and the dashed line represents a 1:1 relationship.

Plant total leaf area simulated by the 3D model was overestimated by 3% compared to leaf area measured from real plants, with an R^2^ of 0.9 and RMSE of 123.5 cm^2^ (**Figure 5A**). In the vertical farm setup without plants, the correlation between the measured and simulated light intensities closely aligned with the 1:1 line, achieving an R^2^ of 0.9 and a root mean square error (RMSE) of 9.2 (**Figure 6**), suggesting reliable simulations of light conditions in the vertical farm by the 3D modelling approach.

**Figure 7:**
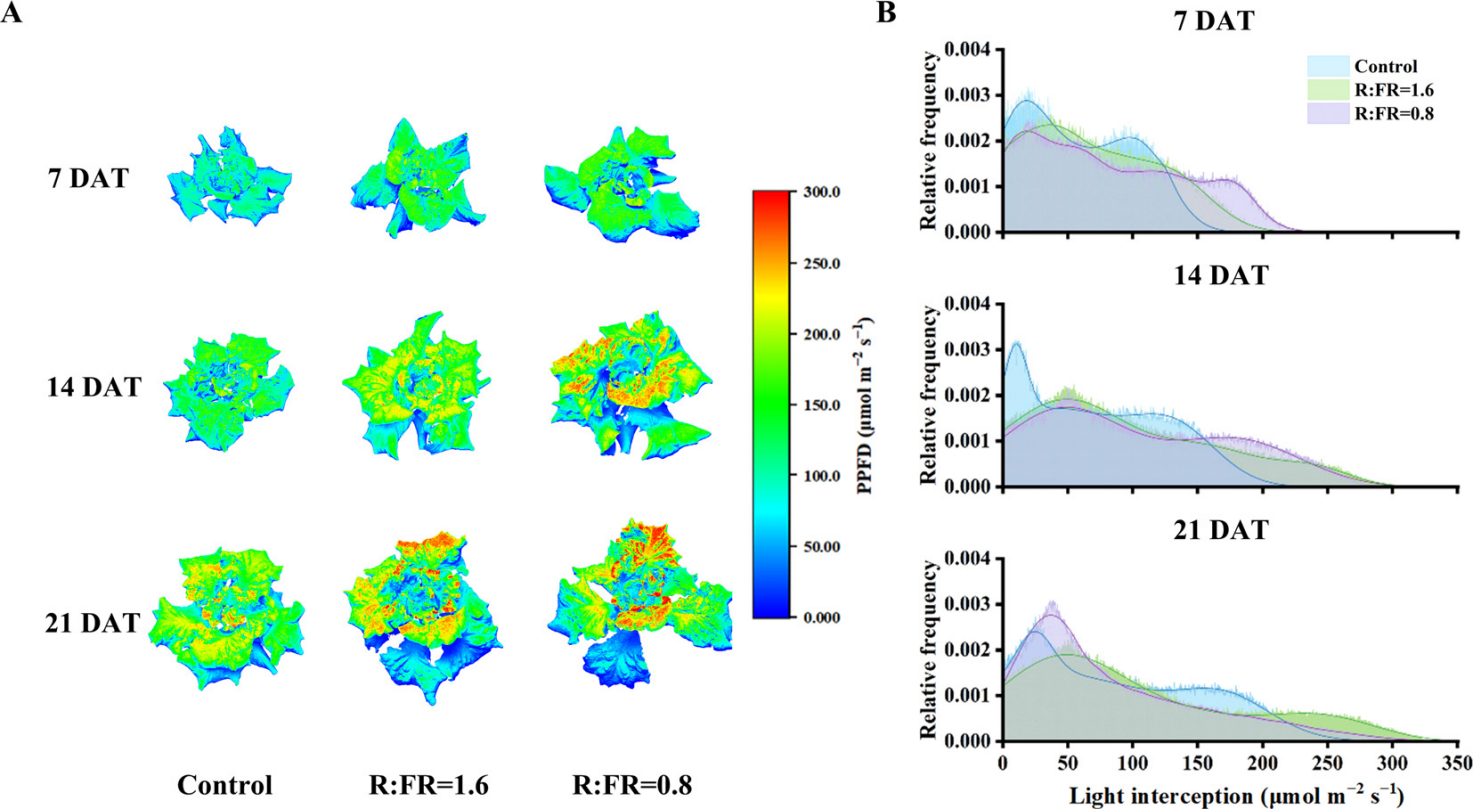
Simulated light interception on 3D-scanned models **(A)** and light intensity distribution at the whole-plant scale **(B)** of lettuce after different FR light conditions at 7, 14 and 21 days.

There were significant differences in plant light absorption under different R:FR treatments. Compared to the control, plant light absorption in treatments with R:FR of 1.6 and 0.8 significantly increased (**Figure 7A**). The absorbed light amount showed maximum values of 178.3, 208.1 and 234.5 μmol s^-1^ m^-1^ under control, R:FR(1.6) and R:FR(0.8) light treatment, respectively, after 7 days of treatment (**Figure 7B**). After 21 days of light treatment, R:FR(1.6) and R:FR(0.8) absorbed significantly more light compared to the control.

There were significant differences in the overall distribution of light across the plant canopy among white light treatment and FR light treatment. After 7 days of treatment, it was showed that most of light intensities range of R:FR(1.6) and R:FR(0.8) was primarily concentrated around high frequency of high light intensity (0 - 180 µmol m^−2^ s ^−1^), while that of the control was observed around relatively low light intensity (0 - 130 µmol m^−2^ s ^−1^). After 14 days and 21 days of treatment, the same pattern of light intensity distribution persisted. R:FR(1.6) and R:FR(0.8) exhibited a high frequency of light intensity distribution at higher levels, compared to the control (**Figure 7B**).

**Figure 8:**
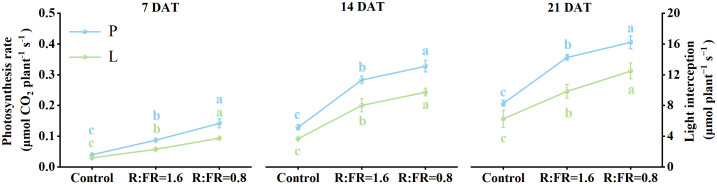
Comparisons of photosynthetic rate and light interception of the scanned parametric model according to the light intensity distribution at the whole-plant scale under different R:FR ratio at 7, 14 and 21 days after treatment. P, photosynthetic rate; L, light interception. The vertical bars indicate SD; n = 3. Different letters at the top of the histogram represent significant differences (p< 0.05).

**Figure 9:**
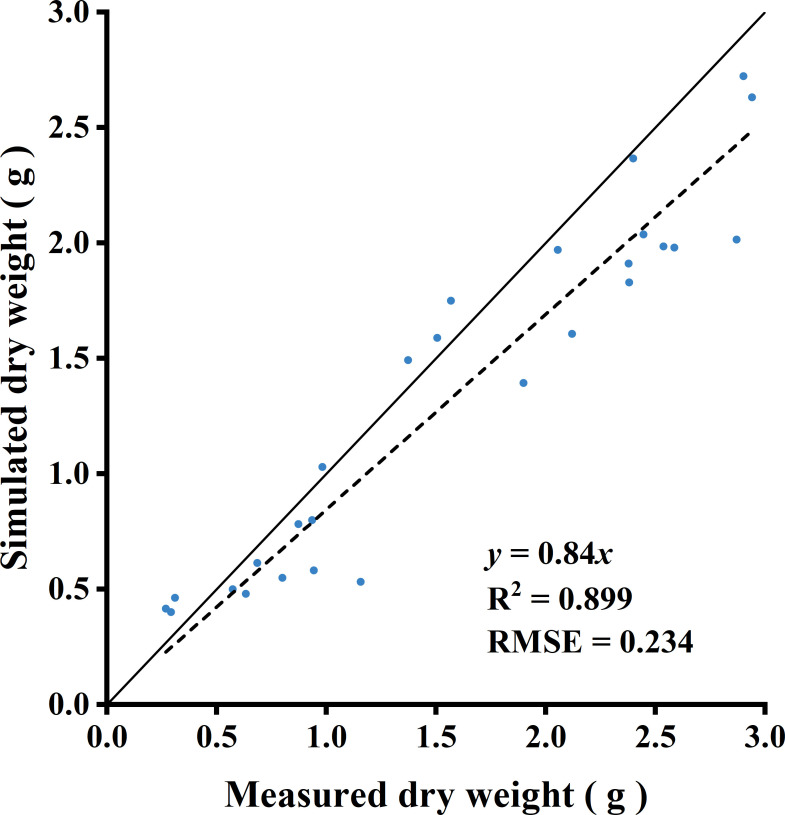
Comparison between measured and simulated dry weight. Three lettuce plants of each treatment were used for measurement. The solid line represents a fitted linear and the dashed line represents a 1:1 relationship.

The light capture of R:FR(1.6) and R:FR(0.8) were significantly higher than the control by 49.1% and 88.3%, respectively (**Figure 8**). Whole-plant photosynthetic rate was the highest in R:FR(0.8), followed by R:FR(1.6), with that of the control exhibiting the lowest plant photosynthetic rate across all growth stages (**Figure 8**). In terms of dry weight simulations, the 3D model slightly underestimated plant dry weight (**Figure 9**). The measured and simulated dry weight corresponded to the 1:1 line with an R^2^ of 0.9 and RMSE of 0.2. The measured and simulated values were matched relatively well when the dry weight was low, and the datapoints scattered with dry weight got higher, indicating that the model captured plant growth relatively well at young developmental stage and underestimated dry weight by up to 28% at later developmental stage (**Figure 9**).

### 3.4 Quantifying the relative contributions of plant morphology and photosynthetic traits on plant light capture and photosynthesis

**Figure 10:**
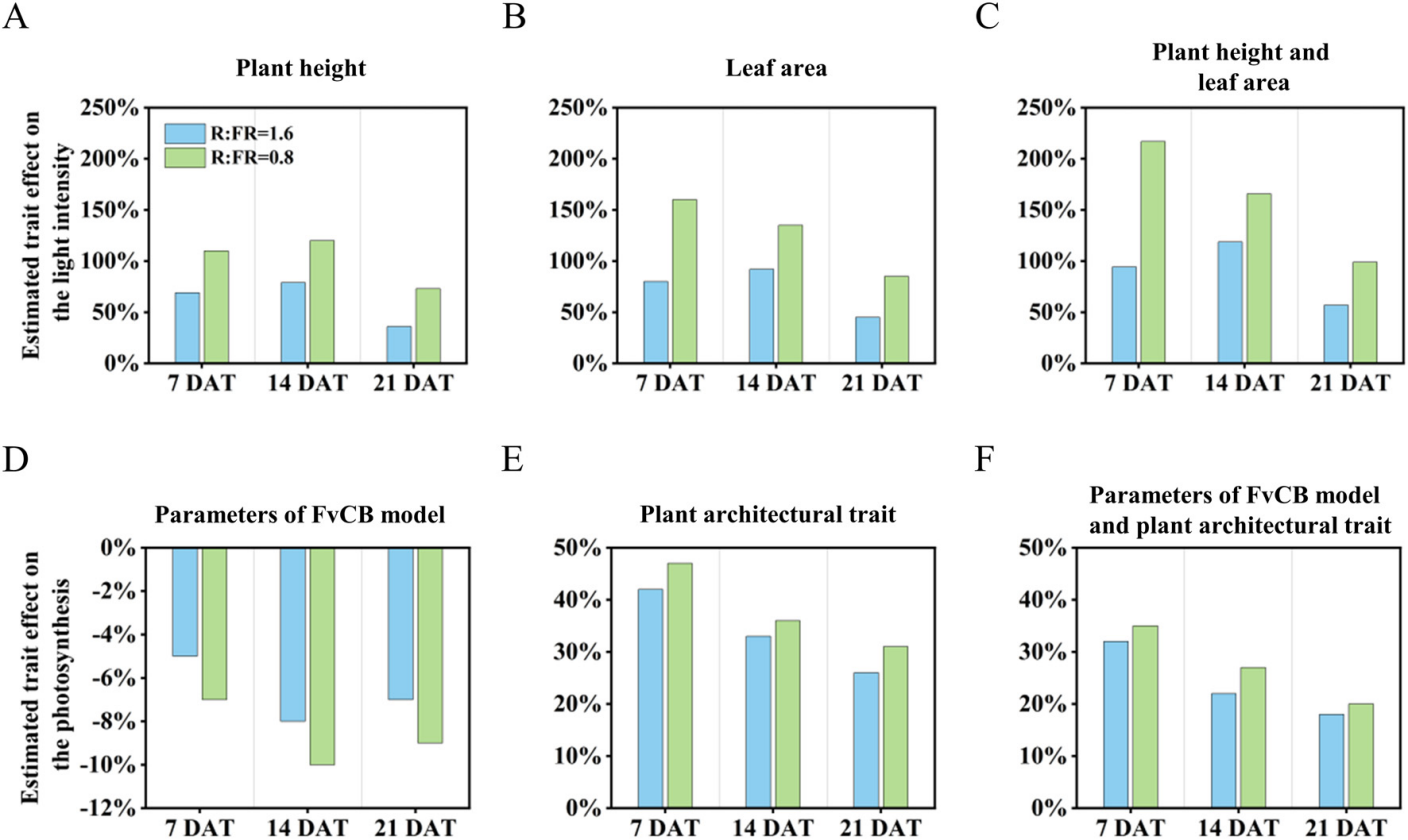
Estimated effects of the indicators of plant height **(A)**, leaf area **(B)**, and combination of the two **(C)** responses to reduced red to far-red ratio on the light intensity for 7, 14 and 21 days after treatment. Estimated effects of the parameters of FvCB model **(D)**, plant architectural trait **(E)**, and combination of the two **(F)** responses to reduced red to far-red ratio on the photosynthesis for 7, 14 and 21 days after treatment.

After 14 days of treatment, the increase in plant height induced by low R:FR ratio caused a minor effect on the fraction of light interception, which increased by 79% and 112% (**Figure 10A**). The increase in leaf area resulting from decreases in R:FR ratio had the greatest impact on the fraction of light interception, which caused an increase by 92% and 135% (**Figure 10B**). Meanwhile, the response of morphological structures (including plant height and leaf area) to reduced R:FR ratio led to an increase in the fraction of light intensity, and this positive effect increased from 1.2- to 1.7-fold when R:FR ratio decreased from 6.4 to 1.6 and 0.8, respectively (**Figure 10C**).

With regard to the effects on the canopy gross photosynthesis, changes in leaf photosynthetic traits caused by reduced R:FR ratio (R:FR(1.6) and R:FR(0.8)) decreased the fraction of the canopy gross photosynthesis by 8.2% and 10.1%, respectively (**Figure 10D**). However, changes in plant architectural trait induced by low R:FR affected canopy photosynthesis in the opposite direction. It was observed that changes of the plant architectural trait as a result of reductions in R:FR(1.6) and R:FR(0.8) increased canopy photosynthesis by 41.9% and 46.8%, respectively, after 7 days of treatment. With increasing days of treatment, these positive impacts diminished, reaching 33.2% and 35.5% after 14 days, and decreasing to only 26.1% and 30.7% after 21 days (**Figure 10E**). In general, when evaluating the combined effects of the two influencing factors, the positive impact of the plant architectural trait offsets the negative impact of the photosynthesis capability of the leaf, and even significantly enhanced the overall photosynthesis of the entire plant. At later period, the reactions to supplemental far-red in R:FR(1.6) and R:FR(0.8) increased the photosynthesis by 18.3% and 20.2% when all trait responses were combined (**Figure 10F**).

## 4 Discussion

### 4.1 Supplemental far-red promoted lettuce growth and enhanced plant height and leaf area

Different light spectra – such as ultraviolet A, green, red, and far-red – have been shown to induce a range of structural, functional, and molecular changes in plants (Klem et al., 2019; Spalholz et al., 2020; Chatterjee et al., 2024). Here, we exposed the Italian lettuce to different levels of supplemental far-red, and showed that in the early stages of lettuce growth, supplementing white LED light with far-red light significantly boosts lettuce growth, with notable increases in plant height and leaf area. This positive impact was increased with the increasing of far-red intensity and the extension of the treatment period, which corresponded with many previous studies. For example, Kalaitzoglou et al. (2019) showed that additional far-red significantly increased the stem elongation and the dimensions of lettuce leaves, thereby increasing the leaf surface for light absorption and accumulating dry matter. Kurepin et al. (2007) demonstrated that far-red increased stem elongation and fresh weight accumulation in sunflower stems. Those responses correspond with the shade avoidance syndrome which is attributed to phytochrome that occurs in a photoequilibrium between the active (Pfr) and inactive (Pr) states. Interception of far-red converted Pfr to the inactive Pr state, lowering the ratio of Pfr that accounts for the total amount of phytochrome, which triggered responses characteristic of avoiding shade, such as steeper leaf inclination angles and increased stem growth (Smith, 1982; Inomata et al., 2005; Franklin, 2008). In general, plants exhibit stem and petiole elongation and changes in leaf arrangements as part of their typical light-seeking behaviors. The Italian lettuce used in this study was a rosette plant which hardly exhibited elongation of central stem. In fact, the increased plant height in the far-red irradiated lettuce plant was largely resulting from the increased leaf elevation angle and elongated lamina length (**Figures 5B, C**), leading to a typical shade-avoidance architecture which often has been found in rosette species such as Arabidopsis (Hu et al., 2021).

### 4.2 Supplemental far-red decreased leaf photosynthetic rate and electron transport rate

So far, there is no consistency in terms of leaf photosynthetic responses to far-red. Early studies demonstrated that far-red light could boost photosynthetic activity in leaves, known as the “Emerson effect” (Govindjee and Hoch, 1964). Generally, Photosystem I (PSI) primarily utilizes far-red light for excitation, while photosystem II (PSII) mainly uses red and blue light; insufficient far-red light leads to imbalanced activation of PSI and PSII, resulting in reduced efficiency of the linear electron transport; therefore, adding additional far-red – when the total amount of incident light is low – promotes the excitation of PSI and enhances the linear electron transport efficiency, and thus photosynthesis (Walters, 2005). Nevertheless, the Emerson effect may diminish with the increasing of incident light intensity. Zhang et al. (2020) showed that adding additional far-red in greenhouse grown roses did not significantly affect leaf photosynthesis. Moreover, Zou et al. (2021) showed that, despite reduced leaf light absorption and lower levels of photosynthetic pigments, photosynthesis in *Lactuca sativa* remained largely unaffected by far-red throughout the growth phase. Here, we found that supplemental far-red decreased leaf photosynthesis in lettuce grown in the vertical farm. In the meantime, photosynthetic rates measured in the light response curve were lower in the R:FR of 1.6 and 0.8 treatment than in tehe control (**Supplementary Figures S4, S5**). Both leaf photosynthetic capacity – i.e. the maximum Rubisco carboxylation rate *V_cmax_* and maximum electron transport rate *J_max_* – and instantaneous net leaf photosynthesis rate under the growth conditions decreased with the increasing of supplemental far-red (**Figure 4**). In line with previous studies, Zhen and van Iersel (2017) found that adding additional far-red with white light reduced photosynthetic rate and chlorophyll content of lettuce. Additionally, we found a rather high curvature factor (*θ*) for the light response curve of electron transport rate (*J*), albeit supplemental far-red did not significantly affect this parameter (**Table 2**). Common values for *θ* are generally between 0.7 – 0.8 (Gu et al., 2012; Yin and Struik, 2015), whereas in our case, *θ* went up to 0.94, almost reaching the highest value that has been reported so far (Ogren, 1993). In general, *θ* describes the shape of the light response of *J* and reflects the amount of absorbed light that has to be dissipated as heat via nonphotochemical quenching (NPQ). Thus, a high *θ* means a low NPQ, which further suggests that the plant does not require much NPQ for photoprotection. Shade-acclimated plants are often found to have high *θ* values (Leverenz, 1988; Ogren, 1993), given that their growth conditions do not require these plants to involve high NPQ to cope with high light stress. Similarly, the lettuce plants in our study were grown under a relatively low light intensity (250 ± 10 μmol m^–2^ s^–1^) – a light intensity that hardly induces high NPQ in plants – thus, having high *θ* values. We conclude that in vertical farms where background photosynthetically active radiation is generally high to ensure crop growth and yield, adding far-red in most cases would hardly bring positive effects on leaf photosynthesis.

### 4.3 Supplemental far-red increased lettuce whole-plant photosynthesis and yield by enhancing canopy light interception

Recently, the positive impact of supplemental far-red light on yield production has been demonstrated in various crops grown in greenhouses and vertical farms. For example, Ji et al. (2019) demonstrated that tomato yield significantly increased under far-red light, and Chen et al. (2022) showed that the enhancement of far-red light increased fruit production of sweet pepper. The improvement in crop yield due to additional far-red light has been attributed to enhanced biomass allocation to fruit, increased canopy light interception, better light distribution, and a larger leaf area (Tan et al., 2022). We also found an enhancement of lettuce growth with supplemental far-red. Both fresh and dry weight of the harvestable part increased with the increasing level of supplemental far-red light, due to increased biomass allocation to aboveground shoots (**Figures 3B-E**). This was in line with a previous study from Jin et al. (2021). Even though the additional far-red light had adverse impacts on leaf photosynthesis, lettuce yield increased with the increasing of far-red light intensity. 3D model simulation results showed that although far-red decreased leaf photosynthesis, leading to reductions in canopy gross photosynthesis (up to 10.2%), far-red induced changes in plant architecture increased canopy light interception, thereby increased canopy gross photosynthesis by up to 46.7% (**Figure 10**). The overall effect of far-red induced changes in both morphological and photosynthetic traits resulted in a positive impact on lettuce whole-plant photosynthesis. The positive impacts of far-red induced morphological changes on plant light capture and growth have been demonstrated in various crops – e.g. tomato, rice, geranium, and snapdragon (Park and Runkle, 2017; Kalaitzoglou et al., 2019; Huber et al., 2024). In general, the increased lamina length and area have been identified as the major reasons for the enhancement of plant light capture by far-red, and the role of internode length on light capture is crop-dependent (Kalaitzoglou et al., 2019; Zhang et al., 2020). The increased leaf elevation angle, however, is often found to reduce plant total light capture while improve canopy light distribution (Zhang et al., 2020, 2021). In our study, supplemental far-red increased lamina area, while also resulted in a more compact plant architecture with steeper leaf elevation angels (**Figures 3G**, **5C**). The increased lamina area positively contributed to plant light capture by up to 150% (**Figure 10B**). The increased plant height – which was a combined result of increased leaf lamina length and elevation angel given the rosette characteristics of the lettuce plant – also brought positive impacts on plant light capture (**Figure 10A**). Furthermore, the more upright plant structure induced by supplemental far-red resulted in a higher frequency of relatively low light intensities captured by the plant (**Figure 7B**), indicating a more evenly distributed light capture at different plant positions. We conclude that far-red induced morphological changes could bring benefit to canopy light capture, thereby increasing canopy gross photosynthesis and yield in lettuce grown in the vertical farm. Additional studies are required to mitigate the negative influence of supplemental far-red on leaf photosynthetic capacity, to further improve crop yield production in vertical farms.

## 5 Conclusion

Supplemental far-red light significantly increased lettuce fresh and dry weight, as well as such morphological traits as leaf area and plant height at different growth stage. However, far-red light significantly reduced leaf photosynthetic capacity and instantaneous net leaf photosynthetic rate. With the 3D modelling approach, we demonstrated that the enhancement of lettuce growth and yield by supplemental far-red was mostly caused by the improved canopy light interception resulted from the morphological changes induced by far-red. We conclude that using supplemental far-red in vertical farms increases lettuce growth and yield, despite its negative impact on leaf photosynthesis. Further studies are needed to understand the mechanisms by which far-red light reduces leaf photosynthetic capacity, which could support the optimization of crop production and the development of cultivars tailored for vertical farming.

## Data Availability

The raw data supporting the conclusions of this article will be made available by the authors, without undue reservation.
