# Supplementary material for: Quantifying the effects of far-red light on lettuce photosynthesis and growth using a 3D modelling approach
Source: Front Plant Sci. 2024 Nov 29;15:1492431. doi: 10.3389/fpls.2024.1492431 (PMC11638671; doi:10.3389/fpls.2024.1492431)
Supplement: Supplementary file 1 [file DataSheet1.docx]

**Fig. S1** The relationship between leaf net assimilation rate and (*I_inc_Φ*_2_/4) of treatment Control (A), R:FR(1.6) (B) and R:FR(0.8) (C). The data was taken from the initial light limiting part of the light curve under low O_2_ backgrounds. Error bars are standard deviations. Equation following the linear regression line for each rank is the regression equation for the organ trait at that rank.


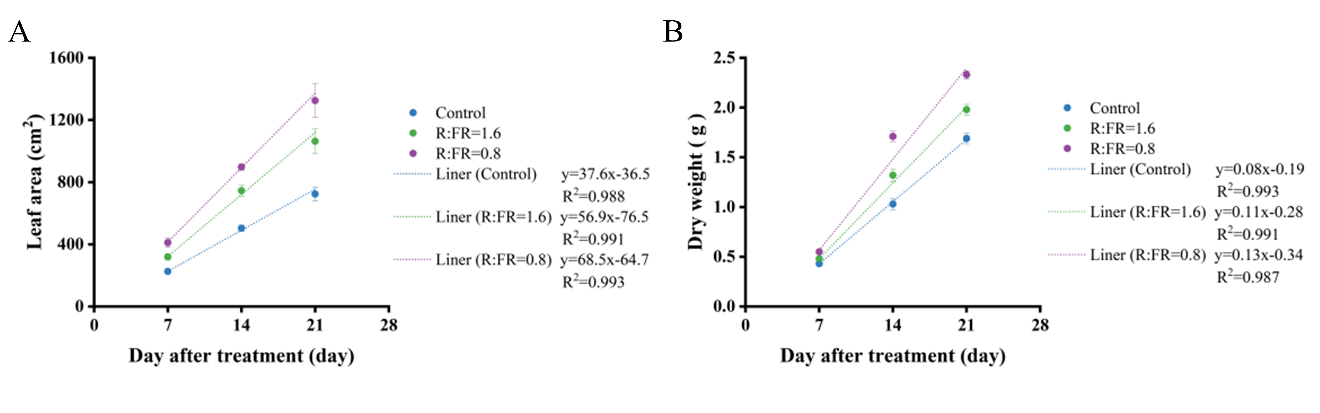


**Fig. S2** The relationship between changes in leaf area and days after treatment (A) and changes in dry weight and days after treatment (B). Error bars are standard deviations. Equation following the linear regression line for each rank is the regression equation for the organ trait at that rank.


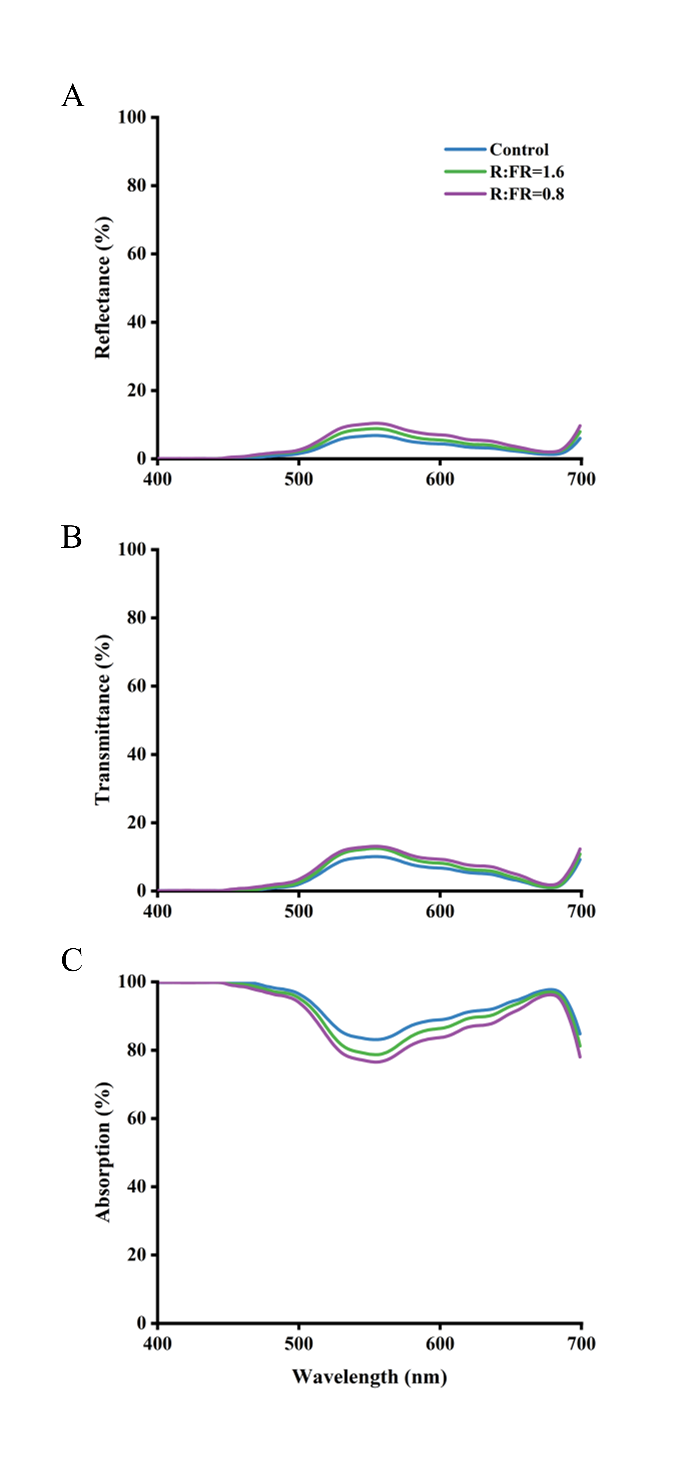


**Fig. S3** Respond of reflectance, transmittance and absorption to FR light in lettuce plants.


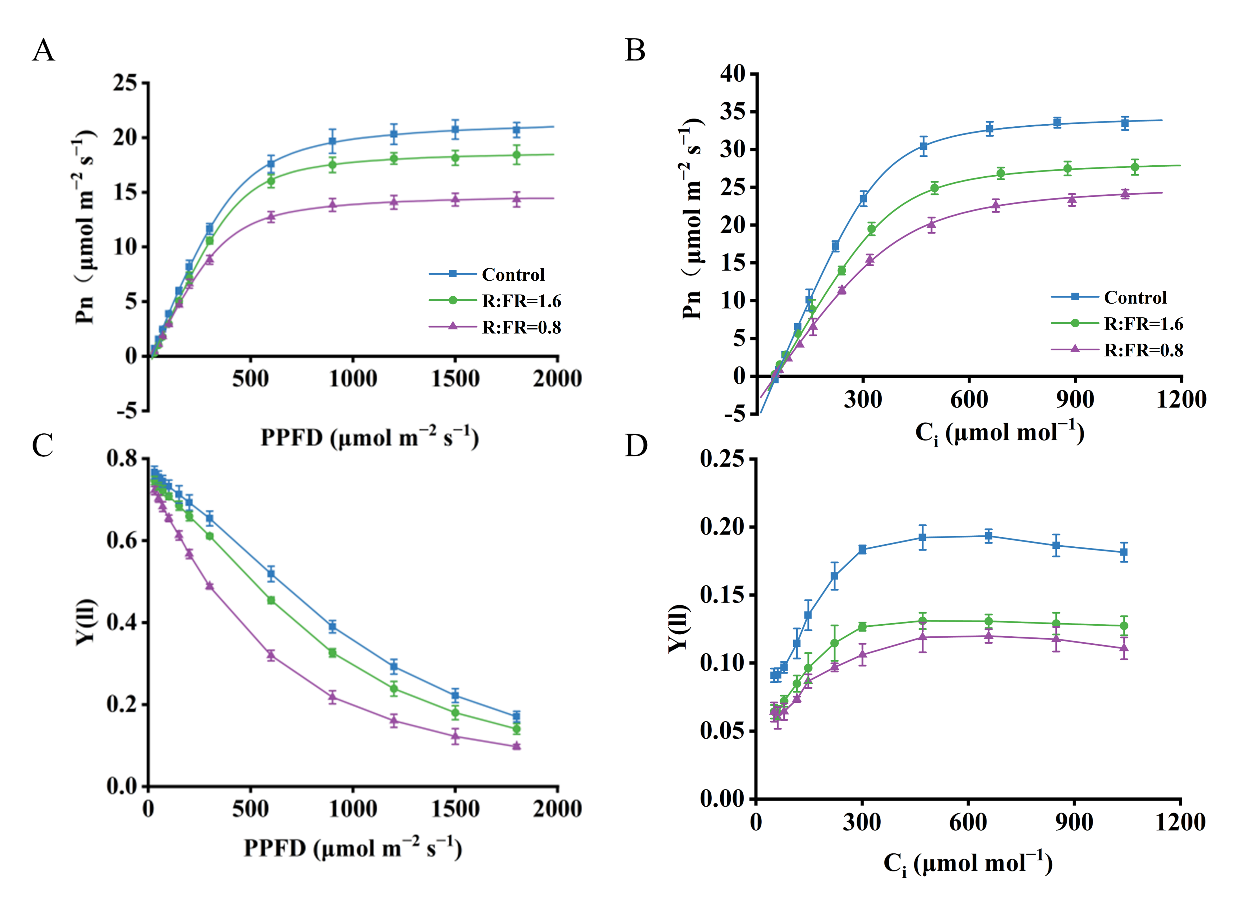


**Fig. S4** Measured net photosynthesis data for leaves in ambient O_2_ backgrounds combined with the data of Y(ll) and the predicted values for the FvCB model, varying PPFD or CO_2_ (A-D). The vertical bars indicate SD; n = 3.


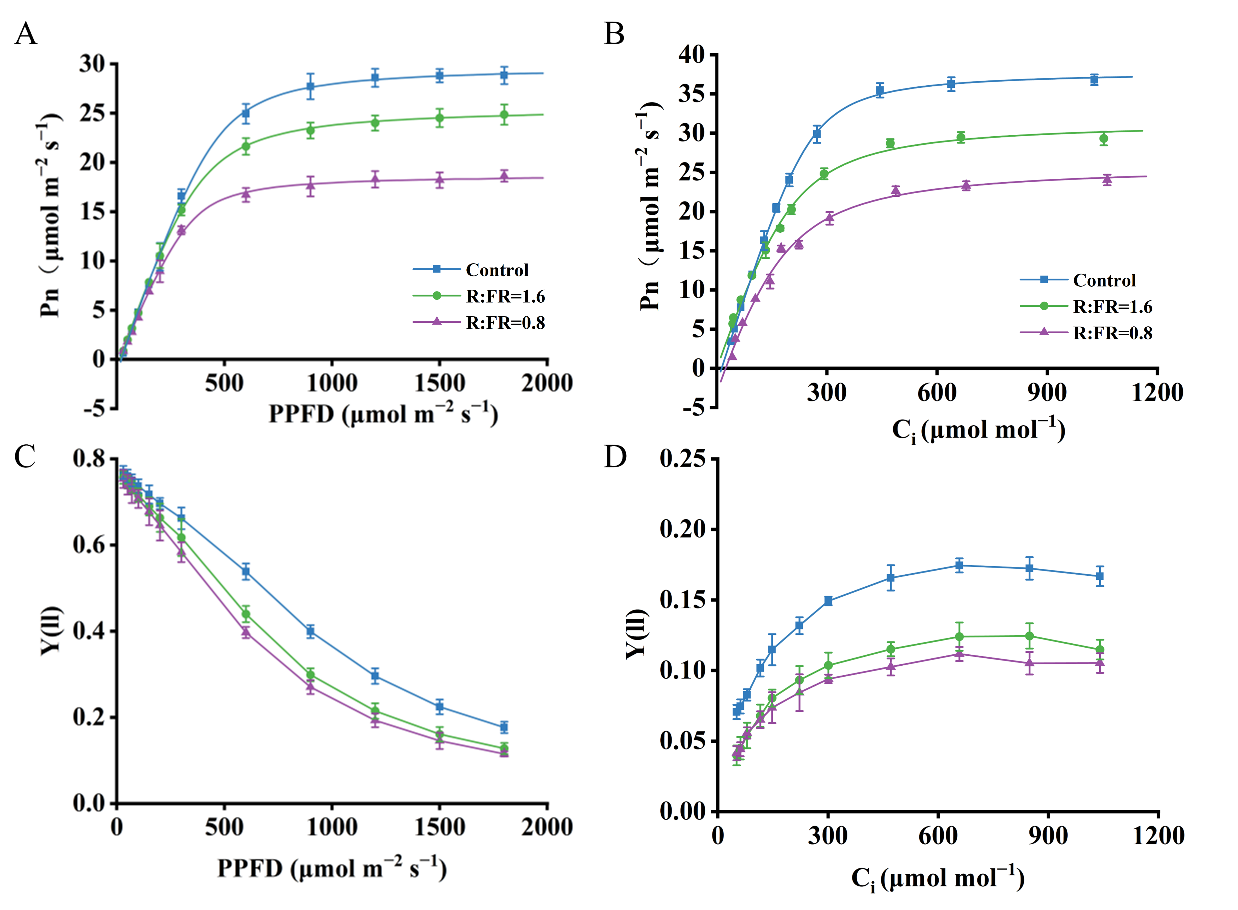


**Fig. S5** Measured net photosynthesis data for leaves in low O_2_ backgrounds combined with the data of Y(ll) and the predicted values for the FvCB model, varying PPFD or CO_2_ (A-D). The vertical bars indicate SD; n = 3.
